# Living Together, Feeling Apart: Loneliness and Living Arrangements Among Older US Immigrants

**DOI:** 10.1093/geroni/igaf122.4156

**Published:** 2025-12-31

**Authors:** Mara Sheftel

**Affiliations:** Rutgers University, New Brunswick, New Jersey, United States

## Abstract

Loneliness tends to increase with age after 50 [3–5] and is a key modifiable risk factor for chronic health conditions in older adulthood [8–11]. Immigrants may be especially vulnerable to loneliness due to disrupted social networks and geographic separation from origin-country ties [12–14]. While older immigrants are more likely than their US-born counterparts to coreside with family [15,16], such arrangements may confer either risk [18] or resilience [17] for mental health, depending on context. This presents an empirical puzzle. This paper investigates how patterns of loneliness and living arrangements vary by country of origin, drawing on Health and Retirement Study (HRS) data for foreign-born Mexican older adults and new data from the New Jersey Population Health Cohort Study (NJHealth) for foreign-born Korean and Indian older adults. Findings reveal distinct patterns compared to US-born counterparts: Indian immigrants are less likely to live alone and show no significant differences in loneliness, Korean immigrants report higher loneliness despite similar living arrangements, and Mexican immigrants are both more likely to report loneliness and less likely to live alone. These results suggest that the relationship between living arrangements and loneliness is not uniform across immigrant groups and may reflect differences in cultural norms, migration histories, and socioeconomic contexts. As the US older immigrant population becomes increasingly diverse [19], understanding group-specific vulnerabilities is critical. This study offers early insights into which older immigrant populations may be at heightened risk of loneliness and how living arrangements intersect with mental health outcomes in later life.

